# Ambient air pollution, temperature and hospital admissions due to respiratory diseases in a cold, industrial city

**DOI:** 10.7189/jogh.12.04085

**Published:** 2022-10-16

**Authors:** Huanhuan Jia, Jiaying Xu, Liangwen Ning, Tianyu Feng, Peng Cao, Shang Gao, Panpan Shang, Xihe Yu

**Affiliations:** 1 School of Public Health, Jilin University, Changchun City, Jilin Province, China; 2 School of Public Administration, Jilin University, Changchun City, Jilin Province, China

## Abstract

**Background:**

The influences of air pollution exposure and temperature on respiratory diseases have become major global health concerns. This study investigated the relationship between ambient air pollutant concentrations and temperature in cold industrial cities that have the risk of hospitalization for respiratory diseases.

**Methods:**

A time-series study was conducted in Changchun, China, from 2015 to 2019 to analyse the number of daily admissions for respiratory diseases, air pollutant concentrations, and meteorological factors. Time-series decomposition was applied to analyse the trend and characteristics of the number of admissions. Generalized additive models and distributed lag nonlinear models were constructed to explore the effects of air pollutant concentrations and temperature on the number of admissions.

**Results:**

The number of daily admissions showed an increasing trend, and the seasonal fluctuation was obvious, with more daily admissions in winter and spring than in summer and autumn. There were positive and gradually decreasing lag effects of PM10, PM2.5, NO_2_, and CO concentrations on the number of admissions, whereas O_3_ showed a J-shaped trend. The results showed that within the 7-day lag period, 0.5°C was the temperature associated with the lowest relative risk of admission due to respiratory disease, and extremely low and high temperatures (<-18°C, >27°C, respectively) increased the risk of hospitalization for respiratory diseases by 8.3% and 12.1%, respectively.

**Conclusions:**

From 2015 to 2019, respiratory diseases in Changchun showed an increasing trend with obvious seasonality. The increased concentrations of SO_2_, NO_2_, CO, PM2.5, O_3_ and PM10 lead to an increased risk of hospitalization for respiratory diseases, with a significant lag effect. Both extreme heat and cold could lead to increases in the risk of admission due to respiratory disease.

Attributable to industrialization and global warming, air pollution has become one of the greatest threats in the current era. Air pollution has an impact on not only climate change but also public and individual health due to its impact on morbidity and mortality [1-3]. The human respiratory tract is highly exposed to the external environment and is more susceptible than other body systems to the influence of external environmental factors, especially air pollutants and air temperature changes [4,5]. The prevalence and mortality of respiratory diseases, as well as the influences of air pollution and meteorological factors on respiratory diseases, have become major global health concerns [6-8].

In China, according to the Health Statistics Yearbook (2020), respiratory diseases are the fourth leading cause of death among urban and rural residents. Epidemiological researches have suggested that the exposure to air pollutants and some meteorological factors have adverse effects on individual health [9], with significant lag and cumulative effects [10,11]; moreover, they are the most important risk factors for respiratory diseases, increasing the number of hospitalizations, and emergency visits as well as mortality due to clinical manifestations [11,12].

Increasing evidence has revealed that particulate matter (PM2.5-10), as a representative air pollutant, can damage the heart and lungs [13,14] and can induce acute myocardial infarction [15]. Exposure to SO_2_, NO_2_ and other air pollutants has also been proven to be closely related to morbidity and mortality due to respiratory diseases [16-20]. Previous researches have shown that short-term exposure to O_3_ or CO was associated with adverse respiratory outcomes, including acute changes in lung function [21,22], asthma, bronchiectasis, pneumonia and the total number of respiratory diseases [23]. Besides, long-term historical O_3_ exposure has also been associated with decreased lung function [24].

Regarding meteorological factors, previous studies have suggested that temperature, humidity, air pressure and other meteorological conditions affect the incidence of respiratory diseases and the number of medical visits [25,26], and exposure to low and high temperatures increase the risk of disease [27]. In addition, in a previous study, the incidence of respiratory diseases caused by high temperature exposure was higher than that caused by low temperature exposure; however, the incidence of respiratory syncytial virus (RSV) infection was related to low temperature exposure [28].

Changchun is the largest automobile manufacturing city in China, with a strong industrial manufacturing capacity. Changchun is located in northeast China, where winters are cold and long, and heating periods are also correspondingly long. The heating energy structure is single, and coal is the main source. The proportion of coal consumption in total primary energy consumption is higher than the national average level. As a result, industrialization and its unique location have led to a poor ambient environment in Changchun, as well as a high incidence of respiratory diseases. However, up to now, there are few researches on the relationship between exposure to air pollutants and meteorological factors with respiratory disease-related admissions in this area.

In this study, through the simultaneous analysis of the hospitalization data of the Hospital Information System (HIS) and the ambient air pollutants and meteorological data, the influence of air pollutant exposure and temperature on the number of admissions for respiratory diseases in Changchun were explored, thus providing theoretical support for the formulation of measures to reduce the incidence of and mortality due to respiratory diseases from an environmental perspective.

## METHODS

### Source of information

#### Data on admissions due to respiratory diseases

The data of inpatients with respiratory diseases in this study was collected from the HISs of four large tertiary hospitals in Changchun from January 1, 2015 to December 31, 2019. The four hospitals are located in the Changchun municipal area, and according to the health statistics yearbook, the service volume of the four hospitals accounts for more than 60% of the total service volume of hospitals in the municipal area. In the 10th Revision of the International Classification of Diseases (ICD-10), the codes J00 ~ J99 represent respiratory diseases, so we collected the data of patients with recorded J00 and J99 codes. Case information included patient age, sex, dates of admission and discharge, diagnosis, etc.

#### Air pollutant and meteorological data

Regional air quality monitoring stations monitor local concentrations of air pollutants in real time and upload the data to the China National Environmental Monitoring Center. In this study, the air quality data in Changchun from January 1, 2015, to December 31, 2019, were collected from the weather website (www.tianqihoubao.com), which tracks historical air quality data of the China National Environmental Monitoring Center. The air quality-related values include the air quality index (AQI) index and the concentrations of six major pollutants, including PM2.5 (μg/m3), PM10 (μg/m3), SO_2_ (μg/m3), NO_2_ (μg/m3), CO (mg/m3) and O_3_ (μg/m3) [11,29]. The AQI is a dimensionless relative value that comprehensively represents the degree of air pollution or the level of air quality, and the higher the AQI is, the more serious the air pollution and the greater the harm to human health. In addition, ambient air quality standards established by the Ministry of Ecology and Environmental of the People’s Republic of China were applied to assess the concentrations of six major air pollutants in Changchun. The standards are shown in **Table 1**.

**Table 1:** Concentrations of six major air pollutants at different air quality levels

Pollutants	Average time	Concentration limits		
**Class I**		**Class II**
SO_2_ (μg/m^3)^	Per 24 h	50		150
NO_2_ (μg/m^3)^	Per 24 h	80		80
CO (mg/m^3)^	Per 24 h	4		4
O_3_ (μg/m^3)^	Maximum 8 h	100		160
PM10 (μg/m^3)^	Per 24 h	50		150
PM2.5 (μg/m^3)^	Per 24 h	35		75

Meteorological data were obtained from the China Meteorological Data Service Center (CMDC, http://data.cma.cn/en), from which we obtained the daily temperature (°C), barometric pressure (hPa) and relative humidity (%) in Changchun in the corresponding period.

#### Data analysis

Descriptive statistical analysis was performed to analyse the relationship among age, gender, and admissions for respiratory diseases, as well as the air pollutants concentrations and meteorological factors from 2015 to 2019. Spearman correlation coefficients were calculated to analyse the correlations between the number of admissions and air pollutant concentrations and meteorological factors.

Time-series decomposition was applied to preliminary analyse the numbers of admissions to explore changes in the number of hospitalized patients and the trend of admissions. Analysis of time series data are usually performed to identify long-term trends, seasonal variations and irregular fluctuations [30-33]. Long-term trend refers to the tendency for a phenomenon to continue to develop and change over a prolonged period of time. Seasonal fluctuation refers to the regular change in the phenomenon associated with seasonal changes. Irregular fluctuation represents the influence of many irrelated factors on time series trends. The addition form of classical decomposition method was used in this research to decompose time series, and the library of StatsModels in Python software (Version 3.2.1, Python Software Foundation, Delaware, USA) was performed for analysis. The expression of addition form was shown in Equation (1).

*Yt = Tt+St+Rt t* = 1, 2, 3, …, n (1)

In which, the definition of *Yt* was time series, *Tt* was long-term trend, *St* was seasonal fluctuation, and *Rt* was irregular fluctuation.

A generalized additive model (GAM) was constructed to explain the complicated nonlinear relationship between the independent variables and dependent variables. GAMs are widely used in environmental epidemiology to explore the relationship between air pollutant exposure and disease-related death or morbidity [34-36]. In this study, the GAM was used to analyse the effects of air pollutants on admissions. Specifically, the lag effect of each 10 μg/m3(0.1 mg/m3 for O_3_) increases in an air pollutant concentration on the number of admissions on the day of admission (lag 0) and over a 7-day lag period (lag 1-7) was examined. Since respiratory disease hospitalization is a low-probability event and its distribution approximately follows a Poisson distribution [37,38], this study established a GAM based on a quasi-Poisson distribution. In the model, the daily number of admissions was considered the outcome, and daily average pollutant concentrations were considered predictors. In addition, in the model, the time trend and meteorological factors were smoothed, and weekday and holiday effects were corrected for. Besides, based on the ICD-10, we extracted clearly classified and representative categories of respiratory diseases, including acute respiratory diseases (J00-J06, J09-J11, J20-J22), chronic respiratory diseases (J31, J32, J37, J40-J47) and pneumonia (J12-J18), to explore the influence of air pollutants on different categories of respiratory diseases. The categories and codes of respiratory diseases analysed are shown in Table S1 in the **Online Supplementary Document**. The expression of DLNM was shown in Equation (2).


*log(Ei) = β (Ci)+ s(Time, df) + s(MT, df) + s(RH, df) + s(MB, df) + DOW + Holiday + α* (2)

In which, the definition of *Ei* is the expected number of admissions with respiratory diseases on day *i. Ci* is the average concentration of air pollutants, and β is the regression coefficients estimated by the model. *Time* is the date variable. *MT, RH* and *MB* are mean temperature, mean relative humidity and mean barometric. The ns was natural smooth spline function. The *df* was degree of freedom, which was determined according to Akaike information criterion (AIC) and previous researches [39-42]. Previous researches have proposed that working days and holiday had influence on the number of admissions [43,44], so the *DOW* and *Holiday* were the effect of weekday and holiday, and both were included in the model as factor variables. The α was the intercept. In the last, the sensitivity analysis was conducted by controlling for different degree of freedom of tine for time and establishing two-pollutant models to exclude the influence of other pollutants to verify the robustness of the model.

A distributed lag nonlinear model (DLNM) was constructed to analyse the effect of temperature on the number of admissions due to respiratory diseases [37]. The core of DLNM is to establish a cross-basis matrix, that is, to select the appropriate basis functions for the two dimensions of explosion-reaction and explosion-hysteresis effect, obtain the cross-basis function by finding the tension product of the two basis functions, and then incorporate the cross-basis matrix into the model for analysis. In this paper, the cross-base matrix was established for number of admission and temperature data, and the number of daily admissions was considered the dependent variable, with adjustments for time trends, the weekday effect, the holiday effect, average relative humidity and barometric pressure. For the cross-base matrix, the maximum number of lag days were set to 7 days and the base function type was a natural spline function. In addition, based on the AIC [38] and Bayesian information criterion (BIC) [45,46], the degree of freedom of the variable basis function and lag basis function were both 2. The quasi-Poisson model was used to fit the relationship between temperature and the number of admissions. In the same way, we also discussed the influence of temperature on different categories of respiratory diseases. The expression of GAM was shown in Equations (3).


*log(Ei) = β basis.temp + ns(Time, df) + ns(RH, df) + ns(MB, df) + ∑ ns(Pl, df) + DOW + Holiday + α* (3)

In Which, *basis,temp* was the cross-basis matrix. The *pl* is the six pollutants in the study. The other variables have the same meaning as the GAM model variables.

Besides, GAM and DLNM models were fitted using the “GAM” and “Dlnm” packages in R software (version 4.1.3, R Foundation, Vienna, Austria).

## RESULTS

### Demographic characteristics of patients admitted due to respiratory diseases

A total of 234 279 patients were hospitalized due to respiratory diseases from 2015-2019. Of these patients, most of the inpatients were male (55.42%) and aged 0-9 years and over 60 years (73.40%). There were 28 349 cases (12.10%) with acute respiratory disease, 39 783 cases (16.98%) with chronic respiratory disease and 109 483 cases (46.73%) with pneumonia. In 2019, 50 640 cases were admitted to hospital with respiratory diseases. The demographic characteristics of the patients admitted due to respiratory diseases are shown in **Table 2**.

**Table 2:** Demographic characteristics of the patients admitted for respiratory disease

Variables	n	%
Gender		
Male	129 830	55.42
Female	10 449	44.58
Age group (years)		
0-9	101 774	43.44
10-19	8793	3.76
20-29	7020	2.99
30-39	10 150	4.33
40-49	13 911	5.94
50-59	22 431	9.58
60-69	28 721	12.26
70 and above	41 479	17.70
Disease subtype		
Acute	28 349	12.10
Chronic	39 783	16.98
Pneumonia	109 483	46.73
Allergic	237	0.10
Year		
2015	43 483	18.56
2016	47 761	20.39
2017	46 015	19.64
2018	46 380	19.80
2019	50 640	21.61
Total	234 279	100.00

### Analyses of air pollutant concentrations and meteorological factors

**Figure 1:**
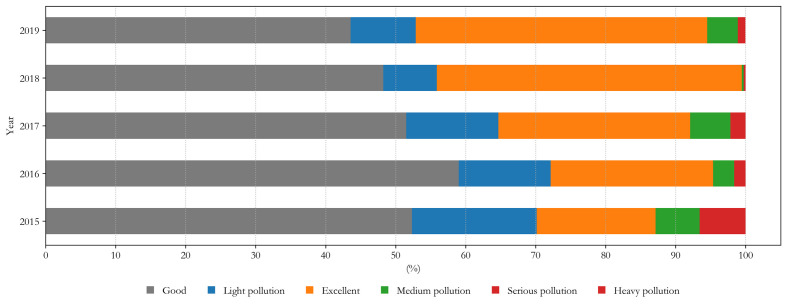
Level of air quality index (AQI) from 2015 to 2019.

The results of the main analyses of air pollutants and meteorological factors in Changchun from 2015 to 2019 are shown in **Table 3**. The results showed that the temperature in Changchun was approximately 7°C, the humidity was approximately 60% and the barometric pressure was 987 hPa. In addition, the air quality gradually improved over time, and the meteorological environment remained stable. In 2019, the proportions of days of PM2.5, PM10, SO_2_, CO, NO_2_ and O_3_ reaching air quality Class I national standard were 55.09%, 34.56%, 88.50%,100%, 99.07%, and 91.35%, respectively. From the perspective of the AQI, the proportion of days with excellent or good air quality between 2015 and 2019 was 81.49% of the total days, and the proportion of light pollution days was 12.21%. The air quality was the worst in 2015, with 12.88% of the days having moderate or worse pollution; only 69.32% of the days had good or excellent air quality. Air quality was the best in 2018, with good or excellent days accounting for the highest proportion, (91.78%) and moderate-pollution or worse days accounting for 0.55%. The AQI values from 2015-2019 are shown in **Figure 1**.

**Table 3:** Average air pollutant concentrations and meteorological factors from 2015 to 2019

Variables	2015	2016	2017	2018	2019
Temperature (°C)	7.25	6.68	7.05	6.92	7.65
Barometric (hpa)	987.08	987.12	986.67	987.12	986.58
Humidity (%)	60.09	63.26	59.60	59.34	57.48
PM2.5 (μg/m^3^)	63.56	44.91	45.03	31.94	37.05
PM10 (μg/m^3^)	102.61	76.45	79.91	61.36	65.00
SO_2_ (μg/m^3^)	32.37	25.46	24.18	13.90	9.95
Co (mg/m^3^)	0.88	0.87	1.13	0.80	0.73
NO_2_ (μg/m^3^)	41.94	36.81	37.02	31.69	32.19
O3 (μg/m^3^)	60.17	59.21	59.02	55.62	56.60
AQI	96.22	75.91	77.97	60.16	66.53

AQI – air quality index

### Correlation analyses of air pollutant concentrations and meteorological factors with hospital admissions

The results showed that except for O_3_, the concentrations of all air pollutants had positive correlations, with correlation coefficients between 0.55 and 0.88. The correlation coefficients of PM2.5 with PM10, SO_2_, CO and NO_2_ were 0.53-0.88, the correlation coefficients of PM10 with SO_2_, CO and NO_2_ were 0.59-0.63, the correlation coefficients of SO_2_ with CO and NO_2_ were 0.66 and 0.53, respectively, and the correlation between CO and NO_2_ was 0.63. For meteorological factors, temperature showed a weak positive correlation with humidity (0.2) and a strong negative correlation with barometric pressure (-0.78), while humidity and barometric pressure showed a weak negative correlation (-0.21). In addition, the number of admissions had a weak negative correlation with O3 concentration (-0.12) and positive correlations with the concentrations of the other air pollutants (0.065-0.17). The number of admissions was negatively correlated with temperature and humidity (-0.23 and -0.12) and positively correlated with barometric pressure (0.15), as illustrated in **Table 4**.

**Table 4:** Correlation of air pollutant concentrations, meteorological factors and hospital admissions (95% CI)

Variables	Admissions	PM2.5	PM10	SO_2_	CO	NO_2_	O_3_	Barometric	Temperature		Humidity
Admissions	1										
PM2.5	0.11 (0.07-0.15)	1									
PM10	0.10 (0.06-0.14)	0.88 (0.87-0.89)	1								
SO_2_	0.17 (0.12-0.21)	0.71 (0.68-0.73)	0.59 (0.56-0.62)	1							
CO	0.07 (0.02-0.11)	0.73 (0.70-0.75)	0.62 (0.59-0.65)	0.66 (0.63-0.68)	1						
NO_2_	0.10 (0.05-0.14)	0.67 (0.64-0.69)	0.6 (0.56-0.63)	0.55 (0.52-0.58)	0.64 (0.61-0.67)	1					
O_3_	-0.13 (-0.18,-0.09)	-0.09 (-0.13,-0.04)	0.07 (0.02-0.12)	-0.37 (-0.41,-0.33)	-0.14 (-0.19,-0.10)	-0.11 (-0.15,-0.06)	1				
Barometric	0.15 (0.11-0.2)	0.44 (0.41-0.48)	0.31 (0.26-0.35)	0.67 (0.65-0.69)	0.39 (0.35-0.43)	0.41 (0.37-0.45)	-0.56 (-0.59,-0.53)	1			
Temperature	-0.23 (-0.27,-0.18)	-0.48 (-0.51,-0.44)	-0.32 (-0.36,-0.28)	-0.79 (-0.8,-0.78)	-0.37 (-0.41,-0.33)	-0.30 (-0.34,-0.26)	0.67 (0.64-0.69)	-0.78 (-0.79,-0.76)	1		
Humidity	-0.12 (-0.17,-0.08)	-0.16 (-0.20,-0.11)	-0.35 (-0.39,-0.30)	-0.25 (-0.29,-0.20)	0.04 (-0.01,0.09)	-0.07 (-0.12,-0.03)	-0.22 (-0.26,-0.17)	-0.21 (-0.25,-0.16)	0.20 (0.16-0.24)		1

CI – confidence interval

### Time-series decomposition analysis of admissions

**Figure 2:**
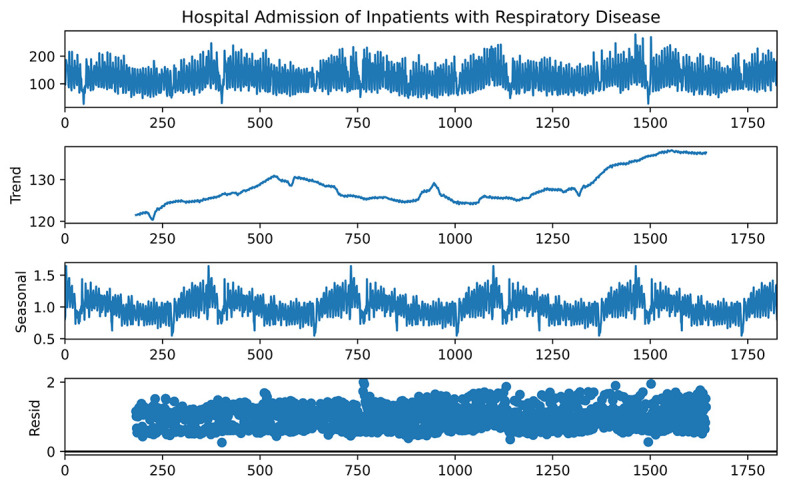
Time-series decomposition analysis of admissions.

The time-series decomposition analysis of the number of admissions from 2015 to 2019 showed that the number of daily admissions due to respiratory diseases had an overall increasing trend. In addition, it had clear seasonal fluctuation, with larger numbers of daily admissions in winter and spring and lower numbers of daily admissions in summer and autumn. The results of time-series decomposition analysis of admissions are shown in **Figure 2**.

### Effects of air pollutant concentrations on the number of admissions

GAMs were constructed to analyse the relationships between air pollutant concentrations and hospital admissions due to respiratory diseases, and the results showed that there were significant correlations between the daily concentrations of air pollutants and the number of admissions.

**Figure 3:**
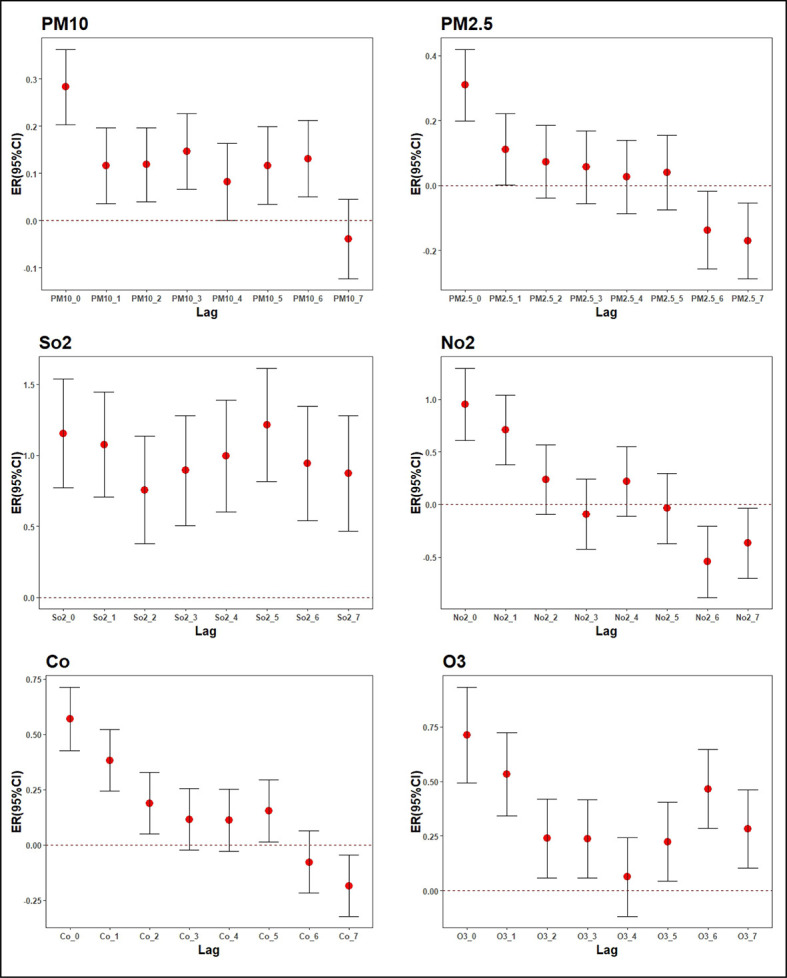
Effects of changes in air pollutant concentrations on the number of admissions.

**Figure 4:**
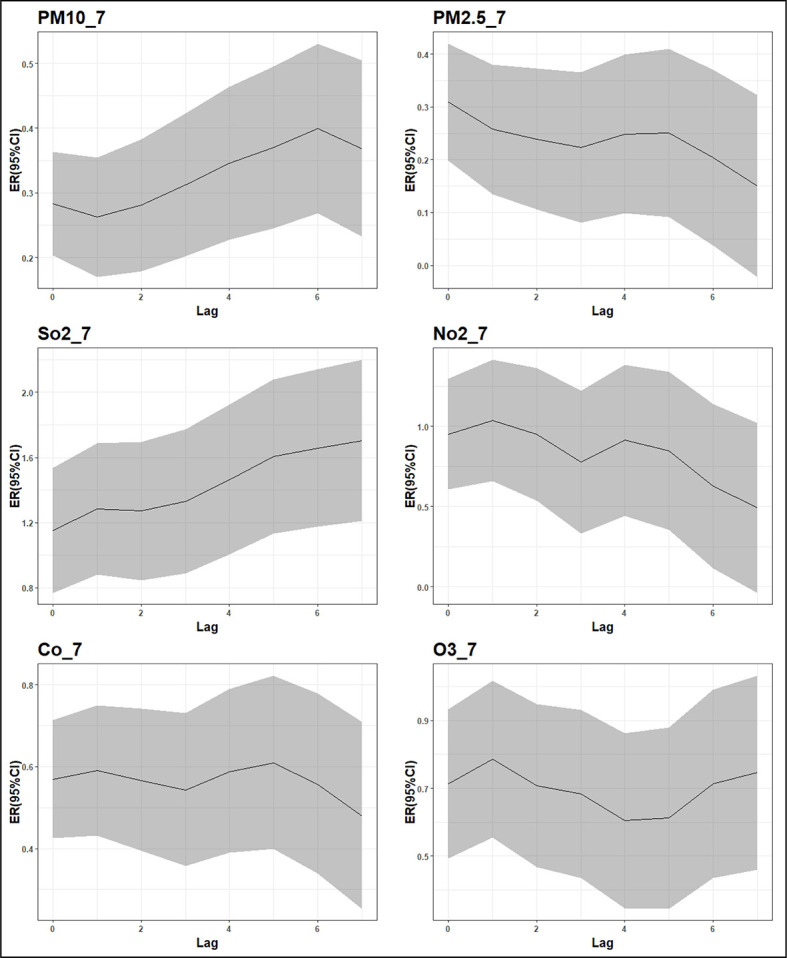
Cumulative lagged effects of changes in air pollutant concentrations on the number of admissions.

Specifically, when the concentration of PM10 increased by 10 μg/m3, the number of admissions due to respiratory diseases increased by 0.28% (95% confidence interval (CI) = 0.20%-0.36%) on lag day 0 and 0.13% (95% CI = 0.05%-0.21%) on lag day 6. When the concentration of PM2.5 increased by 10 μg/m3, the number of admissions increased by 0.31% (95% CI = 0.20%-0.42%) on lag day 0 and -0.17% (95% CI = -0.29%, 0.05%) on lag day 7. When the concentration of SO_2_ increased by 10 μg/m3, the number of admissions increased by 1.15% (95% CI = 0.77%-1.54%) on lag day 0 and 0.87% (95% CI = 0.47%-1.28%) on lag day 7. When the concentration of NO_2_ increased by 10 μg/m3, the number of admissions increased by 0.95% (95% CI = 0.61%-1.29%) on lag day 0 and -0.55% (95% CI = -0.88%, -0.21%) on lag day 6. When the concentration of CO increased by 0.1 mg/m3, the number of admissions increased by 0.57% (95% CI = 0.43%-0.71%) on lag day 0 and -0.18% (95% CI = -0.32%, -0.04%) on lag day 7. When O_3_ increased by 10 μg/m3, the number of admissions on lag day 0 increased by 0.71% (95% CI = 0.49%-0.93%), and the number of admissions on lag day 7 changed by 0.28% (95% CI = 0.10%-0.46%), as described in **Figure 3** and **Table 5**. From the perspective of the cumulative effects of various pollutants, the cumulative effects of PM10 and SO_2_ are gradually enhanced, and the cumulative effects were strongest at 0-6 days and 0-7 days respectively. The cumulative effects of Co and O_3_ tend to fluctuate, whereas the cumulative effects of PM2.5 and NO_2_ are gradually reduced. As shown in the **Figure 4**. Besides, the results of sensitivity analysis showed that the percentage change and 95% CI of the number of admissions with respiratory diseases changed slightly, indicating that the model was relatively robust. As shown in Table S2-S3 in the **Online Supplementary Document**.

**Table 5:** The lag effect of each 10 μg/m3(0.1 mg/m3 for O_3_) increase in air pollutant concentration on the number of admissions (95% CI)

Lagging days	PM10	PM2.5	SO_2_	NO_2_	CO	O_3_
PM10_0	0.28 (0.20-0.36)	0.31 (0.20-0.42)	1.15 (0.77-1.54)	0.95 (0.61-1.29)	0.57 (0.43-0.71)	0.71 (0.49-0.93)
PM10_1	0.12 (0.04-0.20)	0.11 (0.000.22)	1.08 (0.71-1.44)	0.71 (0.38-1.04)	0.38 (0.24-0.52)	0.53 (0.34-0.72)
PM10_2	0.12 (0.04-0.20)	0.07 (-0.04,0.18)	0.76 (0.38-1.13)	0.24 (-0.09,0.57)	0.19 (0.05-0.33)	0.24 (0.06-0.42)
PM10_3	0.15 (0.07-0.23)	0.06 (-0.05,0.17)	0.89 (0.51-1.28)	-0.09 (-0.42,0.24)	0.11 (-0.02,0.25)	0.24 (0.06-0.42)
PM10_4	0.08 (0.00-0.16)	0.03 (-0.09,0.14)	0.99 (0.60-1.39)	0.22 (-0.11,0.55)	0.11 (-0.03,0.25)	0.06 (-0.12,0.24)
PM10_5	0.12 (0.03-0.20)	0.04 (-0.07,0.16)	1.21 (0.81-1.61)	-0.04 (-0.37,0.30)	0.15 (0.01-0.29)	0.22 (0.04-0.40)
PM10_6	0.13 (0.05-0.21)	-0.14 (-0.26,-0.02)	0.94 (0.54-1.35)	-0.55 (-0.88,-0.21)	-0.08 (-0.22,0.06)	0.47 (0.29-0.65)
PM10_7	-0.04 (-0.12,0.05)	-0.17 (-0.29,-0.05)	0.87 (0.47-1.28)	-0.37 (-0.70,-0.04)	-0.18 (-0.32,-0.04)	0.28 (0.10-0.46)

CI – confidence interval

For different disease subtypes, the concentrations of PM10, PM2.5, SO_2_, NO_2_, Co and O_3_ had significant effects on the number of admissions with acute respiratory diseases, the greatest effects were found at lag0, lag0, lag2, lag 2, lag 2 and lag 6, respectively, and except PM2.5, the cumulative effect of pollutants showed an increasing trend in 0-7 days. The concentrations of PM10, SO_2_, CO and O_3_ had significant effects on the number of admissions with chronic respiratory diseases, and the greatest effects were found at lag4, lag7, lag0 and lag0, respectively, whereas the effects of PM2.5 and NO_2_ were not statistically significant. Meanwhile, the cumulative effects of PM2.5 and NO_2_ fluctuated during 0-7 days, and the cumulative effects of other pollutants showed an increasing trend. The concentrations of PM10, PM2.5, SO_2_, NO_2_, CO and O_3_ had significant effects on the number of admissions with pneumonia, and the greatest effects were found at lag0, lag0, lag0, lag2, lag0, lag0, respectively. Besides, except SO_2_, the cumulative effect of pollutants decreased during 0-7 days. The lagged and cumulative effects of pollutants on the disease subtypes is shown in figures S1-S6 in the **Online Supplementary Document**.

### Effects of temperature on the number of admissions

**Figure 5:**
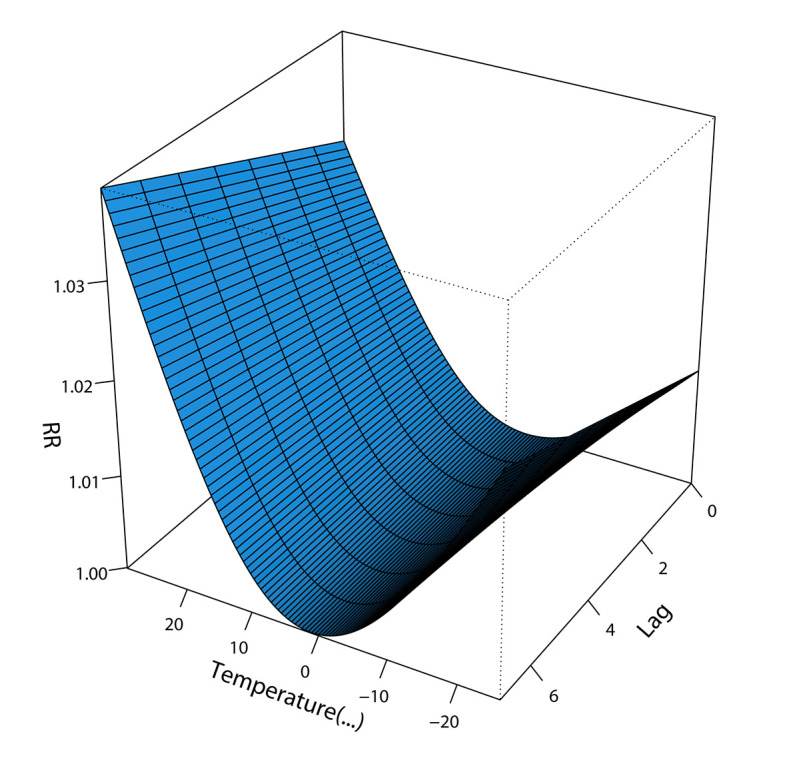
Effects of temperature on admissions.

**Figure 6:**
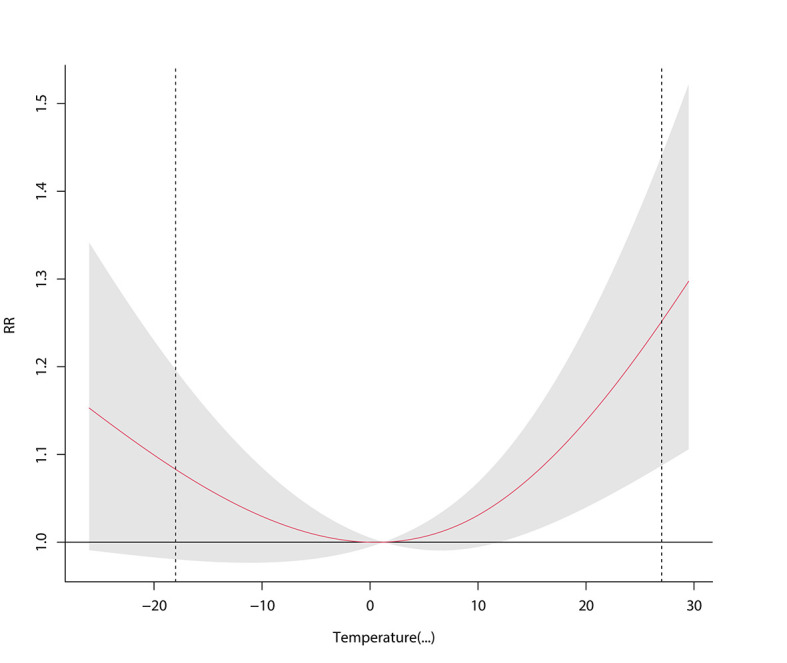
Effects of extreme temperatures on admissions.

The results showed that within 7-day lag period, 0.5°C was the temperature associated with the lowest relative risk of admission due to respiratory disease, and the risk of hospitalization increased significantly under low-temperature and high-temperature conditions. Relative to the temperature associated with the lowest relative risk of admission, extremely low temperature (<-18°C) increased the cumulative risk of hospitalization for respiratory diseases and pneumonia by 8.3% (95% CI = -1.9%-19.6%) and 12.1% (95% CI = 2.2%-23.0%), respectively, within the 7-day lag period. In addition, residents were more sensitive to changes in high-temperature conditions, as the risk of morbidity increased obviously with small temperature changes, and the risk of hospitalization increased with the increase in lag days. Specifically, extreme heat (>27°C) increased the cumulative risk of hospitalization due to respiratory disease and pneumonia by 25.2% (95% CI = 8.7%-44.1%) and by 40.1% (95% CI = 22.2%-60.5%), respectively, within the 7-day lag period. However, the effects of extreme temperatures on acute and chronic respiratory diseases were not statistically significant. As described in **Figure 5**, **Figure 6** and Figure S7-S9 in the **Online Supplementary Document**.

## DISCUSSION

This study included a time-series analysis of changes in the number of hospital admissions due to respiratory diseases in Changchun, an important city in northeast China, from 2015 to 2019. GAMs and DLNMs were applied to analyse the exposure-response relationships, and lag effects of six major air pollutants (PM2.5, PM10, NO_2_, SO_2_, CO and O_3_) and temperature on the number of admissions. This study revealed relationships between environmental factors and the number of hospitalized admissions due to respiratory diseases in a cold and important industrial area.

The results showed that the number of admissions for respiratory diseases increased annually from 2015 to 2019, and the number had increased by 14.30% from 2015 to 2019. In addition, we found that there was an obvious seasonal pattern in the number of admissions for respiratory diseases; that is, there were larger numbers of admissions in winter and spring than in summer and autumn. The main reason is that Changchun is located in northeast China and has cold and long winters. This study and previous studies have proven that lower temperatures increase the risk of respiratory diseases [47,48]. Low-temperature conditions can easily cause temperature inversion, which hinders the diffusion of pollutants [49,50]. In addition, coal combustion is the main heating source in the Changchun area, which leads to increases in pollutant concentrations [51], thus resulting in an increase in the incidence of respiratory diseases. Previous studies have shown that about 30% of atmospheric particulate matter in winter was generated by residents burning coal for heating [52], and more than 400 000 premature deaths have been associated with residential coal combustion [53]. Our results proved strong correlations between temperature and the concentrations of six main air pollutants; that is, the lower the temperature is, the higher the pollutant concentration is. Therefore, we propose that the incidence of respiratory diseases can be effectively reduced by encouraging residents to take effective preventive measures and reduce air pollution by optimizing heating methods [54].

Fortunately, with China's efforts to strengthen the control and prevention of environmental pollution, especially air pollution, health problems caused by air pollution have been received increasing attention [55], and a series of measures to address air pollution have been taken in Changchun. The results showed that from 2015 to 2019, the concentrations of the main air pollutants in Changchun showed decreasing trends each year. The concentrations of PM2.5, PM10, SO_2_, CO, NO_2_ and O_3_ decreased by 41.71%, 36.65%, 69.28%. 16.69%, 23.24% and 5.93%, respectively. AQI, the dimensionless relative value that comprehensively characterizes the degree of air pollution, generally showed a downward trend, with a decrease of 30.86% in 2019 compared with 2015. Moreover, the proportion of days with good or excellent air quality increased from 69.32% to 85.21%. Therefore, significant achievements in reducing air pollutants have been made in Changchun, and the air quality has been gradually improving. However, despite the overall improvement in air quality, the number of admissions showed an increasing trend from 2015 to 2019. This general increase may be related to the increased demand for medical services, excessive demand for medical services [56] and meteorological factors.

The results showed that increased concentrations of air pollutants were significantly associated with an increased number of hospital admissions due to respiratory diseases. When the concentrations of PM10, PM2.5, SO_2_, NO_2_ CO, and O_3_ increased by 10 μg/m3 (0.1 mg/m3 for CO), the maximum percent changes in the number of admissions were 0.28%, 0.31%, 1.21%, 0.95%, 0.57%, and 0.71%, respectively, within 7 days. Previous studies have revealed that PM2.5, PM10 and other particulate matter have significant impacts on the morbidity and mortality of respiratory diseases, especially in infants and children [57]. In addition, a study of 29 European countries found that for every 10 μg/m3 increase in the concentration of PM10, the risk of death due to respiratory diseases increased by 0.58% [58], similar to the results of this study. A study in Shenzhen, China, showed that when PM10, PM2.5, SO_2_, NO_2_, and O_3_ concentrations increased by 10%, the risks of hospitalization for respiratory diseases increased by 0.29%, 0.23%, 0.22%, 0.25% and 0.22%, respectively [11]. Regarding O_3_, a cohort study in the United States demonstrated that long-term exposure to O_3_ increased the risk of respiratory disease-related death in the study population [59]. In particular, the effect of air pollutants on acute respiratory diseases and pneumonia was particularly significant, and there was a cumulative trend of increasing overall. From the aspect of biological mechanisms, scholars [60,61] have noted that exposure to various air pollutants may cause oxidative damage to the respiratory tract, cause oxidative stress in the lungs, and increase the probability of lung inflammation and infection. Therefore, although the air quality level in Changchun is gradually improving, due to its status as a cold, industrial city with a long winter, attention should be given to the control of air pollutants.

Regarding temperature, the results showed that exposure to extreme heat and cold significantly increased the number of admissions for respiratory diseases, with significant lag and cumulative effects. Extreme heat (>27°C) and extreme cold (<-18°C) increased the risk of admissions for respiratory diseases by 25.2% and 8.3%, respectively, within 7 days, and the number of admissions was more sensitive to changes in heat. Besides, this study also showed that extreme temperatures increase the risk of pneumonia. Grigorieva E found that hot weather increased the risk of respiratory disease and death [62], while Ebi K found a stronger correlation between respiratory disease mortality and colder temperatures [63]. From the perspective of biological mechanisms, temperature can directly affect the development of respiratory tract infection by affecting inflammatory pathways or pathophysiological responses, such as vasoconstriction in the mucous membrane of the respiratory tract and suppression of the immune response [64-66]. Besides, sudden temperature changes may lead to bronchospasms and inflammatory changes. In addition, temperature may also indirectly affect predisposing factors of respiratory diseases, such as the transmissibility and outdoor survival time of viruses and bacteria. Relevant studies have shown that the physiological defense function of the upper respiratory tract weakens under low-temperature conditions, and virus infection can inhibit the vitality of ciliated cells and phagocytosis of alveolar macrophages, which in turn leads to the invasion of bacteria, including Streptococcus pneumoniae and Klebsiella pneumoniae, contributing to the high incidence of respiratory diseases under cold conditions [67]. Therefore, it is crucial to increase monitoring and reporting of extreme temperatures and increase awareness about self-prevention measures in residents to reduce the incidence of respiratory diseases.

The study explored the relationships between air pollutants and temperatures with the number of admissions due to respiratory diseases, as well as the exposure-response and hysteresis relationships among them. This study had a large sample size and strict quality control and was scientific and representative. Obviously, there are limitations to this study. First, personal exposure to air pollutants includes ambient exposure and indoor exposure, and indoor exposure data were not available. Therefore, this paper analysed only ambient exposure, which may cause deviations in the results. Second, the characteristics of construction and the environment are different in different regions. This study reflects only the relationship of air pollutants and temperature with the number of admissions due to respiratory diseases in Changchun, but it can provide a theoretical reference for relevant studies in other regions.

## CONCLUSIONS

The study suggests that from 2015 to 2019, respiratory diseases in Changchun showed an increasing trend with obvious seasonality. The increases in SO_2_, NO_2_, CO, PM2.5 and PM10 concentrations resulted in an increase in the number of hospital admissions for respiratory diseases, with a significant lag effect. Besides, both the extreme heat and cold have led to an increase in the number of hospital admissions for respiratory diseases. This study provides a theoretical reference for the formulation of air pollution prevention and control policies and reductions in air pollutants and temperature changes to protect the health of residents.

## Additional material


Online Supplementary Document
