# Supplementary material for: Ambient air pollution, temperature and hospital admissions due to respiratory diseases in a cold, industrial city
Source: J Glob Health. 2022 Oct 16;12:04085. doi: 10.7189/jogh.12.04085 (PMC9569423; doi:10.7189/jogh.12.04085)
Supplement: Online Supplementary Document [file jogh-12-04085-s001.pdf]

ONLINE SUPPLEMENTARY DOCUMENT

Title: Title: Ambient Air Pollution, Temperature and Hospital Admissions due to Respiratory Diseases in a Cold, Industrial City

Authors: Huanhuan Jia, Jiaying Xu, Liangwen Ning, Tianyu Feng, Peng Cao, Shang Gao, Panpan Shang, Xihe Yu

Table S1. The categories and codes of respiratory diseases analysed in this paper.

Categories	Diseases	Codes
Acute respiratory diseases	Acute upper respiratory infections	J00-J06
	Influenza	J09-J11
	Other acute lower respiratory	J20-J22
Chronic respiratory diseases	Chronic rhinitis, nasopharyngitis or pharyngitis	J31
	Chronic nasosinusitis	J32
	Chronic laryngitis or laryngotracheitis	J37
	Chronic lower respiratory diseases	J40-J47
Pneumonia	Pneumonia	J12-J18
Allergic respiratory diseases	Allergic rhinitis	J30.1-J30.4
	Allergic asthma	J45.0-J45.1

Table S2. Sensitivity analysis of effects of changes in air pollutant concentrations on the number of admissions with respiratory diseases when controlling for different degrees of freedom (df) for time. (%, 95% CI)

Variables		lag0	lag1	lag2	lag3	lag4	lag5	lag6	lag7
**PM10**	**df+1**	0.31(0.23,0.39)	0.12(0.04,0.20)	0.12(0.04,0.20)	0.15(0.07,0.23)	0.09(0.01,0.17)	0.12(0.04,0.20)	0.13(0.05,0.22)	-0.04(-0.12,0.05)
	**df**	0.28(0.20,0.36)	0.12(0.04,0.20)	0.12(0.04,0.20)	0.15(0.07,0.23)	0.08(0.00,0.16)	0.12(0.03,0.20)	0.13(0.05,0.21)	-0.04(-0.12,0.05)
	**df-1**	0.26(0.18,0.34)	0.10(0.02,0.18)	0.10(0.02,0.18)	0.13(0.05,0.20)	0.06(-0.02,0.14)	0.09(0.01,0.17)	0.11(0.03,0.19)	-0.06(-0.14,0.02)
**PM2.5**	**df+1**	0.36(0.24,0.48)	0.12(0.01,0.24)	0.08(-0.04,0.19)	0.06(-0.05,0.18)	0.04(-0.07,0.16)	0.05(-0.06,0.17)	-0.14(-0.26,-0.01)	-0.17(-0.29,-0.05)
	**df**	0.31(0.20,0.42)	0.11(0.00,0.22)	0.07(-0.04,0.18)	0.06(-0.05,0.17)	0.03(-0.09,0.14)	0.04(-0.07,0.16)	-0.14(-0.26,-0.02)	-0.17(-0.29,-0.05)
	**df-1**	0.28(0.17,0.39)	0.09(-0.02,0.20)	0.05(-0.06,0.16)	0.03(-0.08,0.15)	0.00(-0.11,0.11)	0.01(-0.10,0.13)	-0.16(-0.28,-0.05)	-0.2(-0.31,-0.08)
**So2**	**df+1**	1.45(1.03,1.87)	1.36(0.96,1.77)	0.91(0.51,1.32)	1.11(0.70,1.53)	1.30(0.87,1.72)	1.50(1.07,1.92)	1.18(0.75,1.61)	1.09(0.66,1.52)
	**df**	1.15(0.77,1.54)	1.08(0.71,1.44)	0.76(0.38,1.13)	0.89(0.51,1.28)	0.99(0.60,1.39)	1.21(0.81,1.61)	0.94(0.54,1.35)	0.87(0.47,1.28)
	**df-1**	0.50(0.17,0.83)	0.48(0.16,0.79)	0.23(-0.10,0.55)	0.28(-0.05,0.61)	0.30(-0.03,0.63)	0.43(0.10,0.77)	0.24(-0.09,0.58)	0.18(-0.15,0.51)
**No2**	**df+1**	0.99(0.64,1.34)	0.78(0.44,1.12)	0.25(-0.09,0.58)	-0.09(-0.42,0.25)	0.26(-0.08,0.60)	-0.02(-0.36,0.32)	-0.54(-0.88,-0.20)	-0.36(-0.70,-0.03)
	**df**	0.95(0.61,1.29)	0.71(0.38,1.04)	0.24(-0.09,0.57)	-0.09(-0.42,0.24)	0.22(-0.11,0.55)	-0.04(-0.37,0.30)	-0.55(-0.88,-0.21)	-0.37(-0.70,-0.04)
	**df-1**	0.89(0.55,1.24)	0.66(0.34,0.99)	0.20(-0.13,0.53)	-0.13(-0.46,0.20)	0.18(-0.15,0.51)	-0.07(-0.41,0.26)	-0.58(-0.92,-0.25)	-0.41(-0.74,-0.08)
**Co**	**df+1**	0.67(0.52,0.83)	0.45(0.30,0.60)	0.20(0.06,0.34)	0.12(-0.02,0.27)	0.13(-0.01,0.28)	0.17(0.03,0.31)	-0.07(-0.22,0.07)	-0.19(-0.32,-0.04)
	**df**	0.57(0.43,0.71)	0.38(0.24,0.52)	0.19(0.05,0.33)	0.11(-0.02,0.25)	0.11(-0.03,0.25)	0.15(0.01,0.29)	-0.08(-0.22,0.06)	-0.18(-0.32,-0.04)
	**df-1**	0.50(0.36,0.64)	0.33(0.19,0.46)	0.14(0.01,0.28)	0.07(-0.07,0.20)	0.06(-0.08,0.20)	0.10(-0.04,0.24)	-0.13(-0.27,0.01)	-0.23(-0.37,-0.10)
**O3**	**df+1**	0.71(0.49,0.93)	0.53(0.34,0.72)	0.24(0.05,0.42)	0.23(0.05,0.41)	0.04(-0.14,0.23)	0.21(0.03,0.40)	0.46(0.28,0.64)	0.28(0.09,0.46)
	**df**	0.71(0.49,0.93)	0.53(0.34,0.72)	0.24(0.06,0.42)	0.24(0.06,0.42)	0.06(-0.12,0.24)	0.22(0.04,0.40)	0.47(0.29,0.65)	0.28(0.10,0.46)

**df-1**	0.58(0.37,0.79)	0.44(0.26,0.63)	0.17(-0.01,0.35)	0.17(-0.01,0.34)	-0.01(-0.18,0.17)	0.15(-0.03,0.33)	0.38(0.21,0.56)	0.21(0.03,0.39)

df – degree of freedom. CI–confidence interval

Table S3. Sensitivity analysis of effects of changes in air pollutant concentrations on the number of admissions with respiratory diseases on lag0 when combed two pollutions.

**Variables**	**PM10**	**PM2.5**	**So2**	**No2**	**Co**	**O3**
**+PM10**	-	-0.04(-0.24,0.15)	0.71(0.29,1.13)	0.4(0.00,0.81)	0.44(0.24,0.64)	0.62(0.40,0.84)
**+PM2.5**	0.31(0.17,0.45)	-	0.82(0.37,1.26)	0.52(0.00.04,1)	0.84(0.57,1.12)	0.64(0.41,0.86)
**+So2**	0.22(0.13,0.31)	0.19(0.06,0.32)	-	0.54(0.13,0.95)	0.52(0.33,0.71)	0.78(0.56,1.00)
**+No2**	0.23(0.14,0.33)	0.19(0.04,0.35)	0.83(0.37,1.28)	-	0.71(0.48,0.95)	0.82(0.59,1.04)
**+Co**	0.01(-0.01,0.22)	-0.25(-0.46,-0.04)	0.20(-0.31,0.70)	-0.42(-0.98,0.13)	-	0.62(0.40,0.84)
**+O3**	0.25(0.17,0.33)	0.26(0.15,0.37)	1.29(0.90,1.67)	1.13(0.78,1.47)	0.52(0.38,0.67)	-

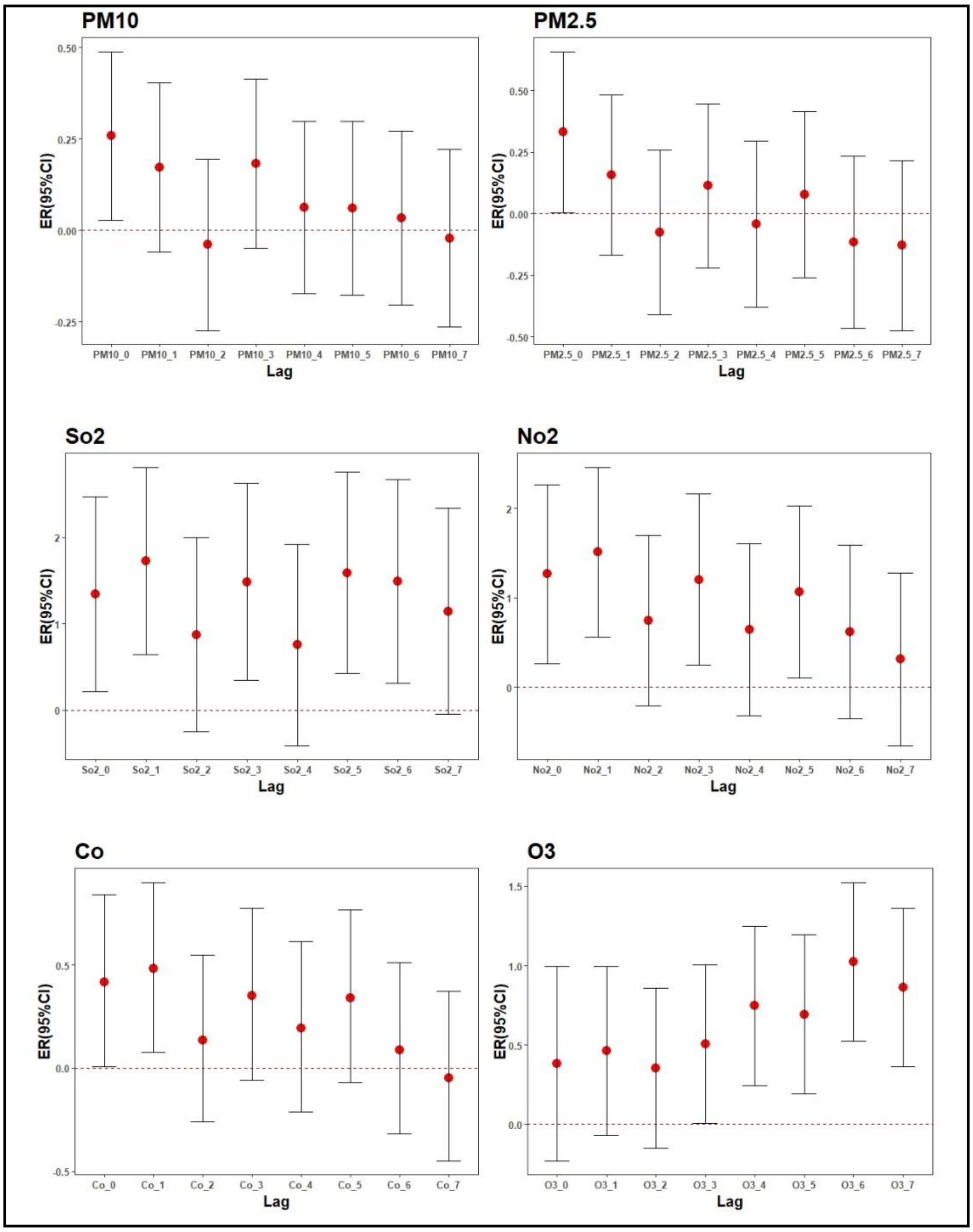

Figure S1. Effects of changes in air pollutant concentrations on the number of admissions with acute respiratory diseases.

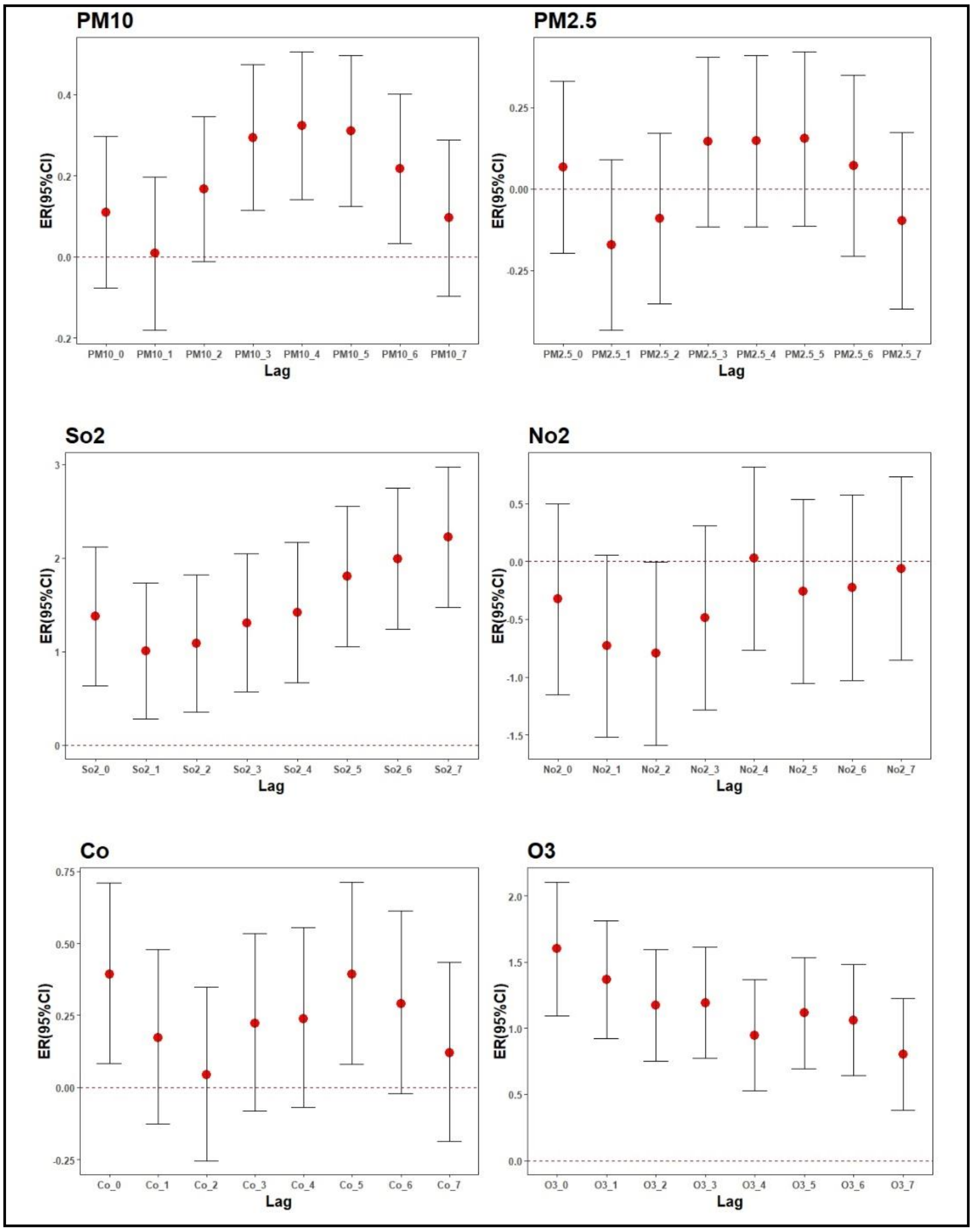

Figure S2. Effects of changes in air pollutant concentrations on the number of admissions with chronic respiratory diseases.

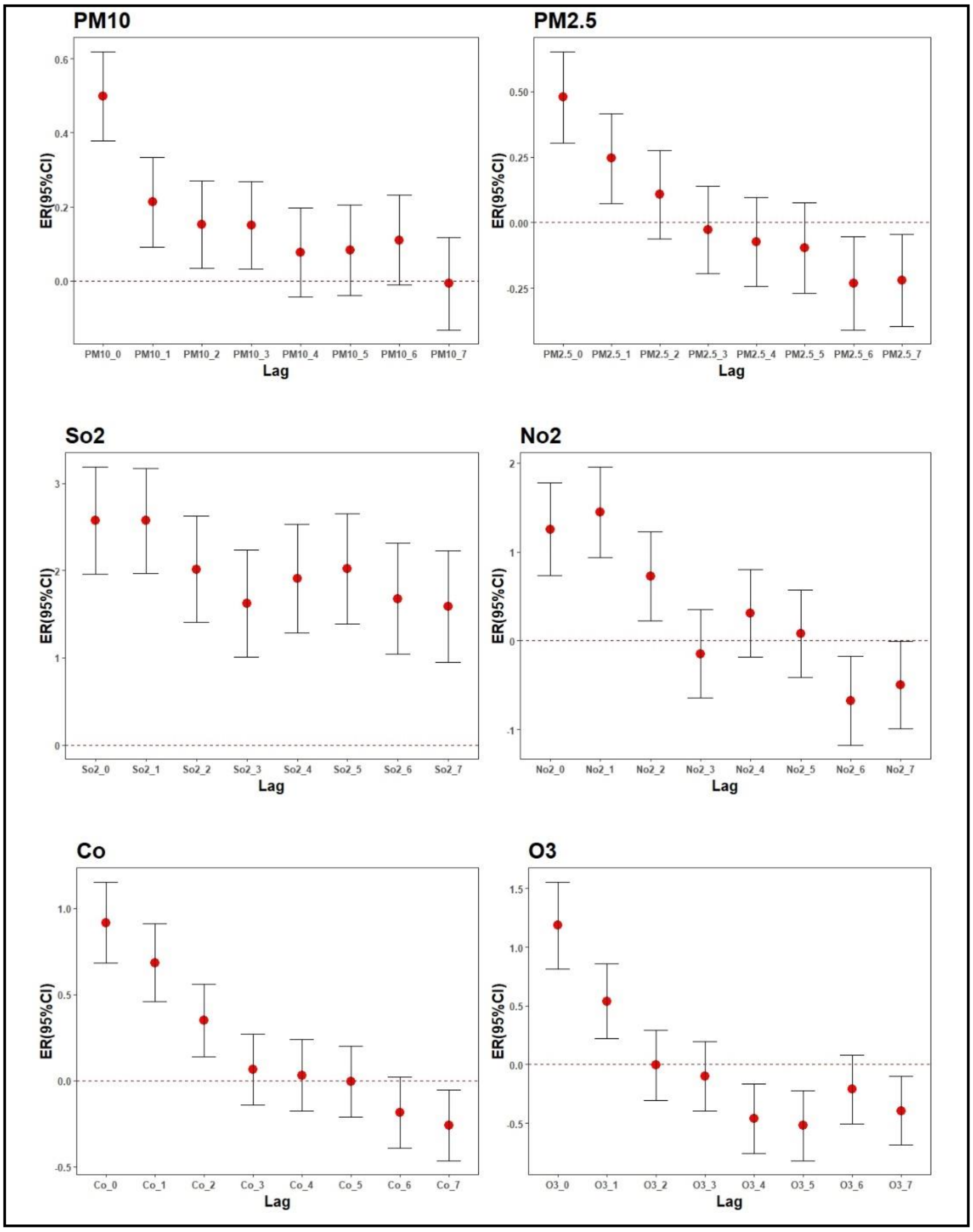

Figure S3. Effects of changes in air pollutant concentrations on the number of admissions with pneumonia.

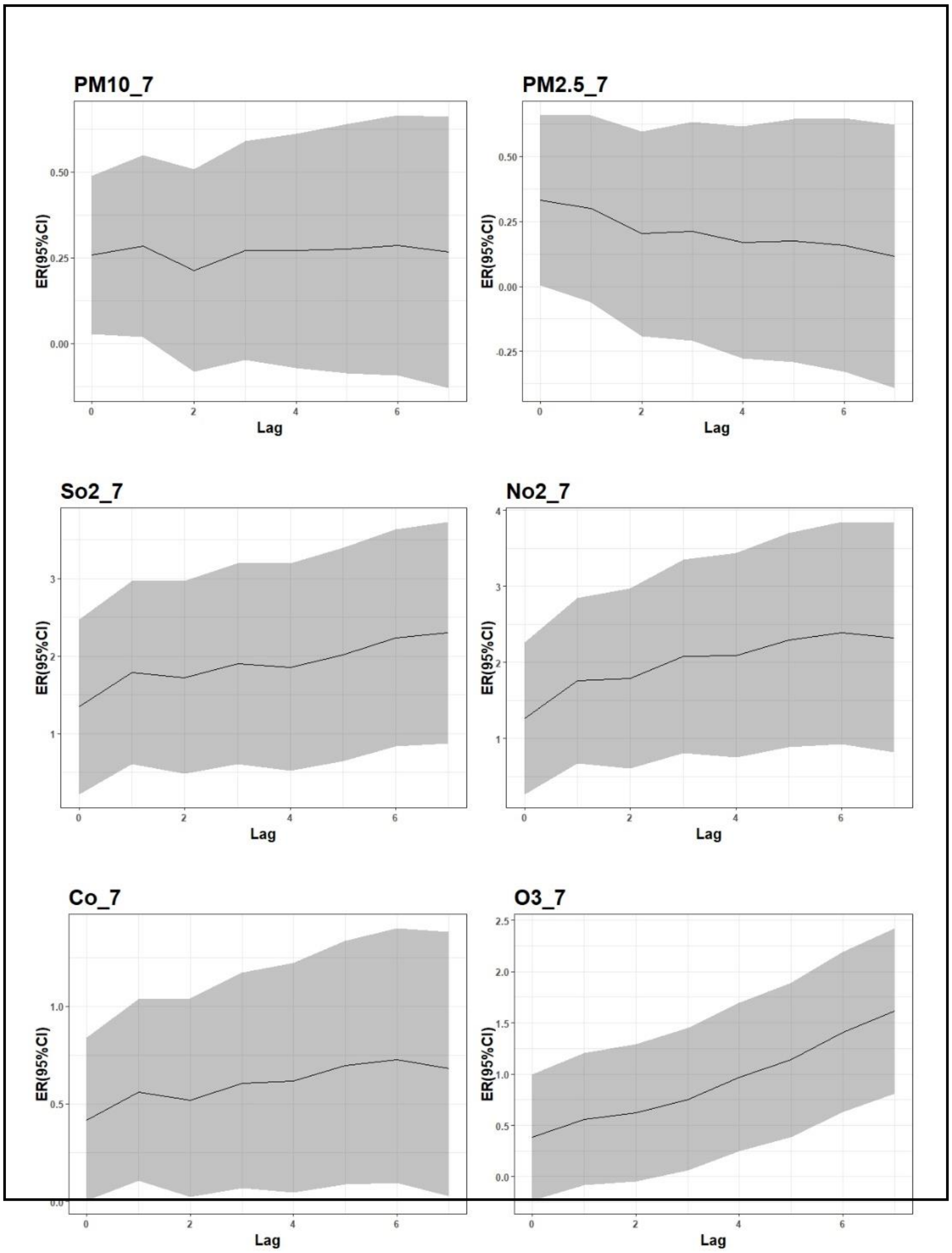

Figure S4. Cumulative lagged effects of changes in air pollutant concentrations on the number of admissions with acute respiratory diseases.

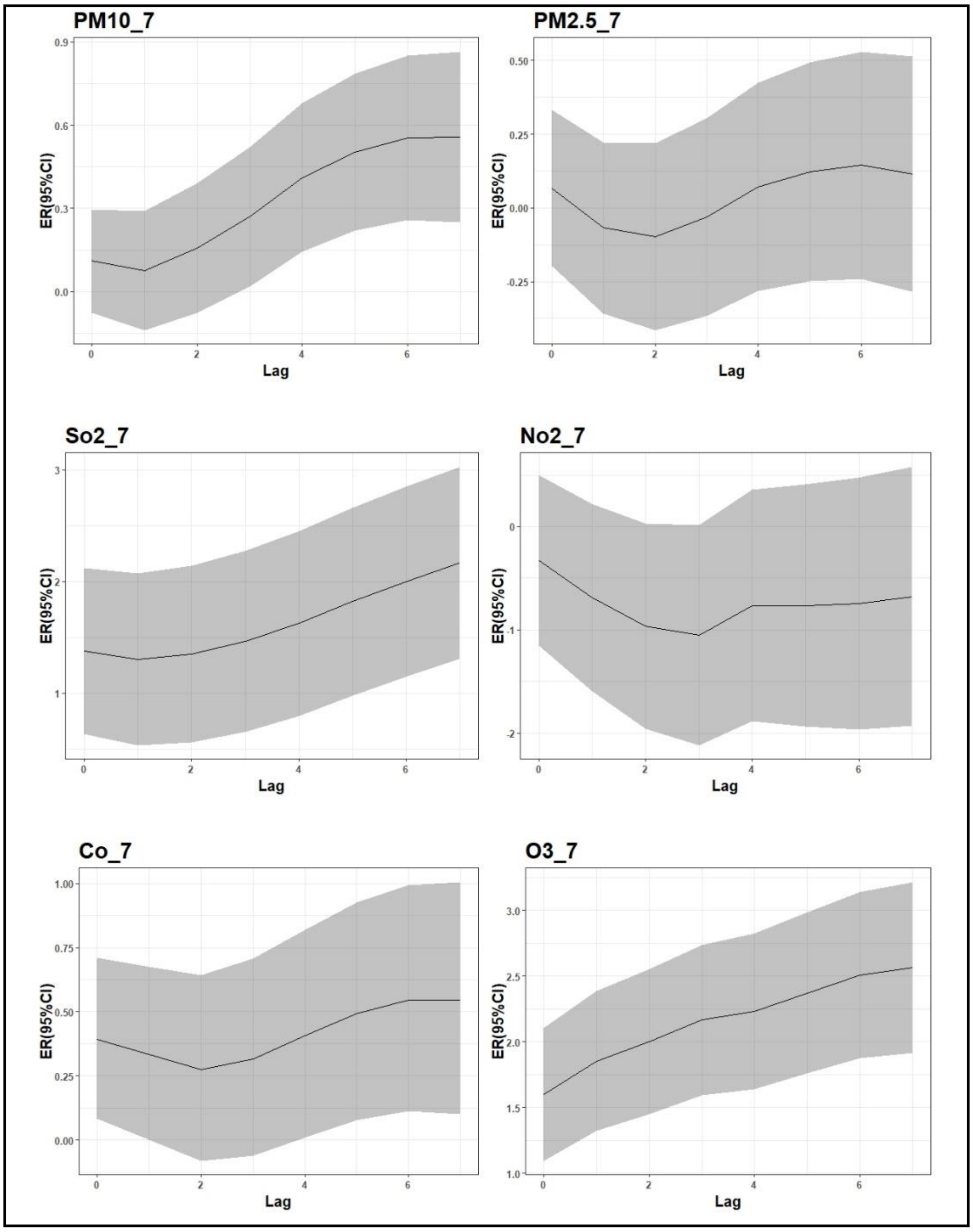

Figure S5. Cumulative lagged effects of changes in air pollutant concentrations on the number of admissions with chronic respiratory diseases.

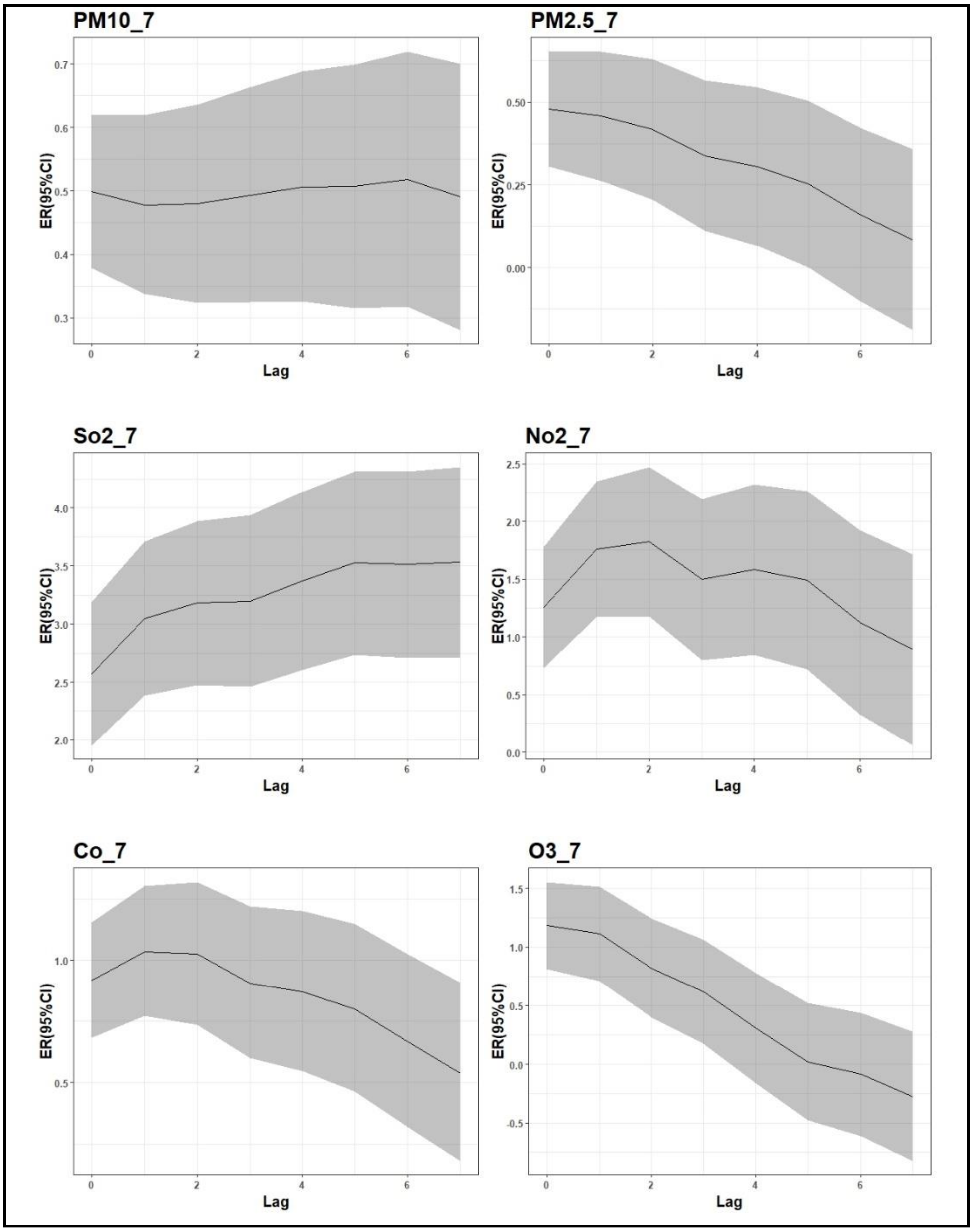

Figure S6. Cumulative lagged effects of changes in air pollutant concentrations on the number of admissions with pneumonia.

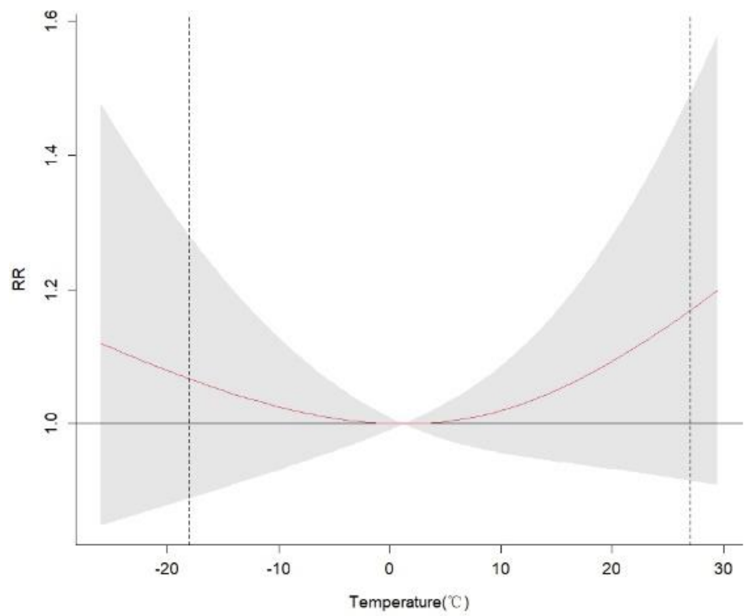

Figure S7. Effects of temperature on admissions with acute respiratory diseases.

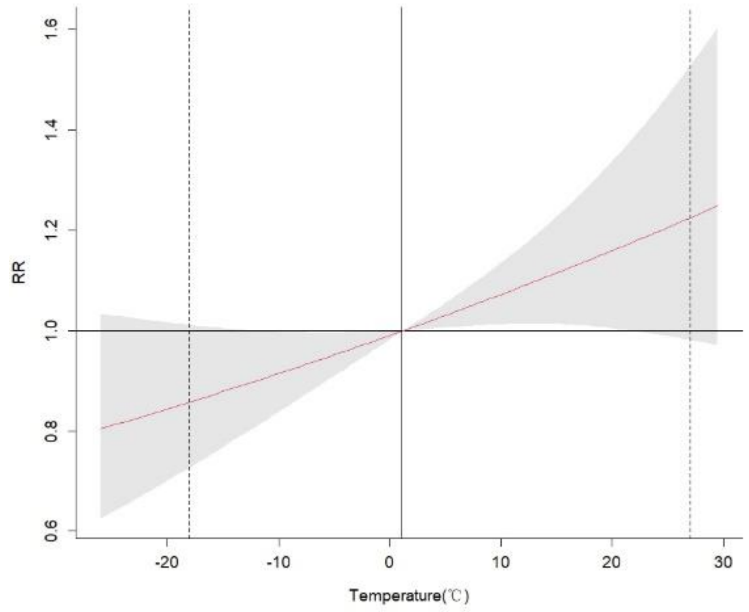

Figure S8. Effects of extreme temperatures on admissions with chronic respiratory diseases.

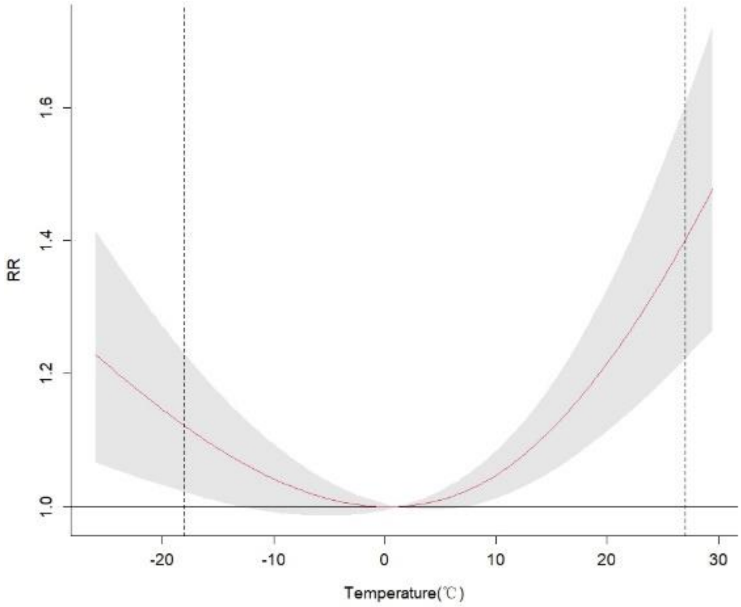

Figure S9. Effects of extreme temperatures on admissions with pneumonia.
